# Chronically Implanted Microelectrodes Cause *c-fos* Expression Along Their Trajectory

**DOI:** 10.3389/fnins.2019.01367

**Published:** 2020-01-10

**Authors:** Patrick Pflüger, Richard C. Pinnell, Nadja Martini, Ulrich G. Hofmann

**Affiliations:** ^1^Section for Neuroelectronic Systems, Clinic for Neurosurgery, Medical Center – University of Freiburg, University of Freiburg, Freiburg im Breisgau, Germany; ^2^Faculty of Medicine, University of Freiburg, Freiburg im Breisgau, Germany

**Keywords:** *c-fos*, NeuN, GFAP, inflammation, striatum, brain implant, ED1, polymer probe

## Abstract

When designing electrodes and probes for brain–machine interfaces, one of the challenges faced involves minimizing the brain-tissue response, which would otherwise create an environment that is detrimental for the accurate functioning of such probes. Following the implantation process, the brain reacts with a sterile inflammation response and resulting astrocytic glial scar formation, potentially resulting in neuronal cell loss around the implantation site. Such alterations in the naïve brain tissue can hinder both the quality of neuronal recordings, and the efficacy of deep-brain stimulation. In this study, we chronically implanted a glass-supported polyimide microelectrode in the dorsolateral striatum of Sprague–Dawley rats. The effect of high-frequency stimulation (HFS) was investigated using *c-fos* immunoreactivity techniques. GFAP and ED1 immunohistochemistry were used to analyze the brain-tissue response. No changes in *c-fos* expression were found for either the acute or chronic stimulus groups; although a *c-fos* expression was found along the length of the implantation trajectory, following chronic implantation of our stiffened polyimide microelectrode. Furthermore, we also observed the formation of a glial scar around the microelectrode, with an accompanying low number of inflammation cells. Histological and statistical analysis of NeuN-positive cells did not demonstrate a pronounced “kill zone” with accompanying neuronal cell death around the implantation site, neither on the polymer side, nor on the glass side of the PI-glass probe.

## Introduction

When an electrode is implanted into the brain, numerous mechanisms are involved in the wound-healing process (Polikov et al., 2005; Biran et al., 2007). Microglia act as “*first responders*” and form the main cellular components in this acutely disturbed environment (Davalos et al., 2005). Their roles involve the removal of blood, debris, and pathogens from the implantation site through cytotoxic means (Polikov et al., 2005), and later during the chronic response, the formation of the glial scar (Röhl et al., 2007). Activated astrocytes are later involved with the reactive gliosis representing a frustrated phagocytosis to remove the foreign body (Reier, 1986; Turner et al., 1999; Polikov et al., 2005; Biran et al., 2007; Leach et al., 2010).

While the functionality of recording and stimulating electrodes are generally favorable in the short term, a degradation in the signal can occur during chronic timescales due to both the neuronal cell loss (the so-called “kill zone”) and the encapsulation of the implant by a glial scar formation (Edell et al., 1992; Liu et al., 1999; Biran et al., 2005). This process may be prolonged, depending on various factors including the initial tissue injury, and the long-term stability of the electrode (Campbell and Wu, 2018).

To alleviate the brain-tissue response, numerous approaches have been made, including alterations in the electrode design (Hofmann et al., 2006), material (Csicsvari et al., 2003; Kipke et al., 2003), coating (Ludwig et al., 2006; He et al., 2007), and implantation techniques (Kim et al., 2004; Wise et al., 2004). To this end, we have conducted a study aimed at examining both the effects of high-frequency stimulation (HFS) in the dorsolateral striatum using microelectrodes and the brain-tissue response in rodents for up to 10 weeks. The targeted brain area features somatotopically organized corticostriatal connections (Voorn et al., 2004) and has already served as model region to highlight the neurochemical effects of HFS (Hiller et al., 2007; Xie et al., 2014). Post-mortem immunohistochemistry was used to probe neuronal (using NeuN as a neuronal marker) activation by the expression of *c-fos* (Dragunow and Faull, 1989; Bullitt, 1990; Herrera and Robertson, 1996; Wilson et al., 2002; Shehab et al., 2014), astrocytic activity by glial fibrillary acidic protein (GFAP), and microglia activity by anti-CD68 (ED1), in order to determine the inflammatory reaction to the chronic implantation of the microelectrode (Turner et al., 1999; Grill et al., 2009; McConnell et al., 2009a; Beck et al., 2010).

## Materials and Methods

### Ethics Statement

All procedures involving animals and their care were conducted in conformity with relevant institutional guidelines in compliance with the guidelines of the German Council on Animal Protection. Protocols were approved by the Animal Care Committee of the University of Freiburg under supervision of the Regierungspräsidium Freiburg (approval G13/97) in accordance with the guidelines of the European Union Directive 2010/63/UE.

### Electrode Assembly

A 12-μm-thick, 380-μm-wide polyimide microelectrode (IMTEK; Freiburg University) as described in Böhm et al. (2019) was superglued (Renfert Dental, Hilzingen, Germany) to a 125-μm glass rod prior to implantation to provide accurate positioning and rigidity to the otherwise flexible probe (Richter et al., 2013). The probe’s shaft contains 12 recording sites (15 μm × 15 μm) and four stimulation sites (50 μm × 50 μm). A large circular aperture, surrounded by a 300-nm-thick platinum ring forms the tip of the shaft.

### Handling, Surgery, and Recovery

**FIGURE 1:**
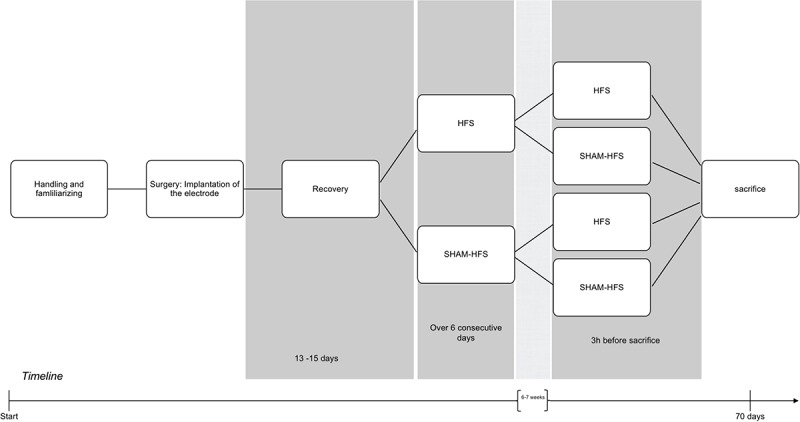
Graphical depiction of the experimental period, including handling, surgery, recovery, and the chronic/acute stimulation sessions.

Prior to surgery, all rats underwent several days of handling in order to familiarize them with the experimenter and test apparatus (see Figure 1 for an experimental timeline).

**FIGURE 2:**
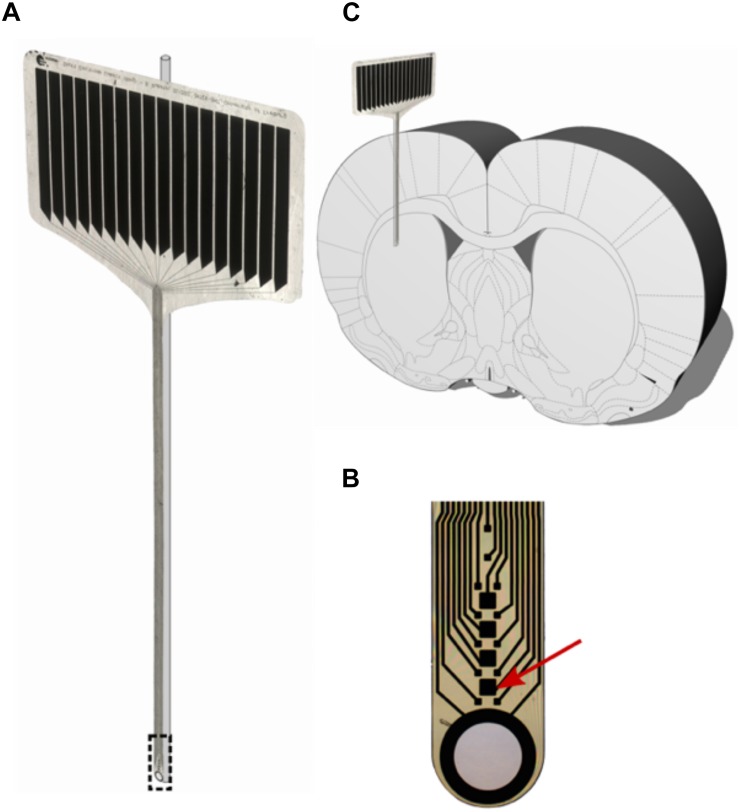
**(A)** 16-contact polyimide micro probe (IMTEK; Freiburg University) with glass rod. **(B)** Magnified tip of the micro probe, with the red arrow pointing at one stimulating contact (50 μm × 50 μm). **(C)** Stiffened probes were implanted parallel to the rat brain’s midline with the polymer facing laterally and the glass rod medially.

Female Sprague–Dawley rats (290–330 g; *n* = 15) were anesthetized with oxygen (0.15 L/min) and isoflurane (AbbVie, United States); the latter of which was initially set to 4% and gradually lowered to 1.5% after placing the animal into the stereotaxic frame (David Kopf, United States). Animal breathing, reflexes and level of anesthesia were monitored throughout the duration of the surgery.

During surgery, animals were implanted with the electrode-glass assemblies in the left dorsolateral striatum (AP: +0.4, ML: +3.6; from Bregma, DV: -3.7 from dura mater) (Paxinos et al., 1985). For this process, a hole was drilled at the electrode site, after which dura was resected using a fine needle. The electrode was subsequently lowered manually at a rate of approximately 200 μm/s, and the skull aperture around the implanted electrode was filled with bone wax. Once in place, the electrode was fixed to a nearby stainless-steel screw anchor (0–80 × 1/8; Plastics One) using a two-compound dental cement (Palapress; Heraeus Holding GmbH; Germany). An additional four screw anchors were used to attach a 3D-printed headstage socket around the electrode assembly (Pinnell et al., 2016) using five stainless steel screws (0–80 × 1/8; Plastics One). An upward-facing Omnetics connector was attached to the electrode assembly, and the headstage was filled with dental cement.

Following surgery, animals were pair-housed, utilizing a sealable headsocket (Pinnell et al., 2016), and were given 13–15 days recovery. Animals were allowed access to food and water *ad libitum*, and were housed under a 12-h light–dark cycle, at 22°C and 40% humidity.

### Stimulation

Following recovery, all animals underwent two stimulation sessions each, which were spaced apart by 6–7 weeks. The first (chronic) stimulation session took place over six consecutive days, and the second (acute) stimulation session took place 3 h before the animals were euthanized (Figure 1); this approach was made for the purposes to obtain a stable *c-fos* expression Dragunow and Faull, 1989). Animals were divided into four groups, on the basis of having received stimulation or sham stimulation at either of these sessions (see Table 1). The stimulation parameters were set to the following: 130 Hz biphasic rectangular pulses, 60 μs pulse width/phase, 400 μA constant current intensity, and 5-min duration using a tethered stimulation system (AlphaLab SNR System, Alpha Omega GmbH, Germany). The geometrical area of stimulating iridium-oxide microelectrode contacts (Mottaghi et al., 2015) was 2500 μm^2^, yielding a charge per stimulating phase of 24 nC/ph and a total stimulating charge of 960 μC/cm^2^. Sham-stimulated animals had underwent the same procedure as their stimulated counterparts (attachment of tether, etc.), but with the absence of electrical stimulation.

**TABLE 1:** Organization of animal groups.

	**Acute session**	**Chronic session**
*n* = 4	STIM	STIM
*n* = 3	SHAM	STIM
*n* = 5	STIM	SHAM
*n* = 3	SHAM	SHAM

### Euthanasia and Histology

Following testing, chronically implanted rats were euthanized with an overdose of isoflurane and perfused transcardially with 4% formaldehyde solution (PFA in phosphate buffer). Their brains were removed, post-fixed in PFA for 7 days, and stored in 30% sucrose until cutting them in coronal sections (20 μm) along the probe’s implantation trajectory with a cryostat. The sections were collected on glass and stored at −20°C until further processing.

### *c-fos* and NeuN Immunofluorescence Staining

The *c-fos* immunoreactivity was visualized using a double-label immunofluorescent staining for *c-fos* and neuronal nuclei (NeuN). Coronal brain sections were processed and incubated overnight with a polyclonal rabbit anti-*c-fos* antibody (sc-52, Santa Cruz Biotechnology, Santa Cruz, CA, United States, diluted 1:100) (Shehab et al., 2014). After rinsing in phosphate-buffered saline (PBS), sections were incubated with a fluorescent donkey anti-rabbit IgG conjugated with Alexa Fluor 647 (Abcam, Burlingame, CA, United States, diluted 1:1000). Sections were rinsed again in PBS, blocked with 10% normal donkey serum (NDS), and incubated for 3 h with a polyclonal mouse anti-NeuN antibody (Anti-NeuN, Millipore Cooperation, Burlington, MA, United States, diluted 1:100) (Mullen et al., 1992). After rinsing in PBS, sections were incubated with a fluorescent donkey anti-mouse IgG conjugated with Alexa Fluor 488 (Abcam, Burlingame, CA, United States, diluted 1:1000). Finally, sections were rinsed again in PBS, mounted with DAPI-Fluoromount G (Southern Biotechnology Associates, Inc., Birmingham, AL, United States) and stored at 4°C.

### Counting of *c-fos*/NeuN-Positive Cells

**FIGURE 3:**
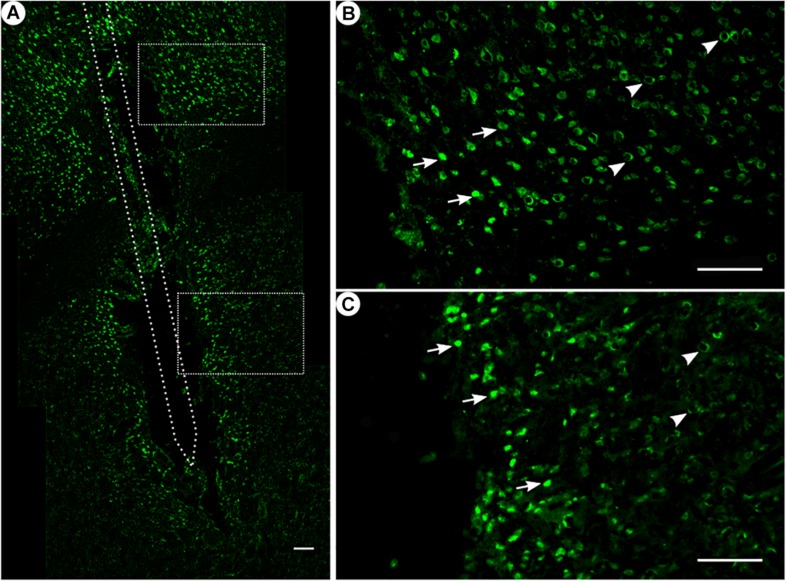
NeuN immunoreactivity. **(A)** Overview of an exemplary corticostriatal area as composite of a series of coronal sections. **(B,C)** Corresponding magnified pictures from panel **(A)**, with white arrows pointing at high NeuN immunoreactivity, and white arrowheads pointing at low NeuN immunoreactivity. Scale bar = 100 μm.

For quantitative analysis, six sections from each animal were used for counting the *c-fos*/NeuN+ cells along the implantation trajectory. Images of stained sections were taken using a Zeiss microscope equipped with a ProgRes camera, along with ProgRes CapturePro 2.7 software (Carl Zeiss, Germany, Jenoptik, Germany). We created composites of the coronal sections using the ImageJ plugin “stitching” (Preibisch et al., 2009), while the brightness and contrast were adjusted as necessary. Using the ImageJ software “cellcounter,” we first quantified the number of NeuN+ cells per box (Figure 3) (100 μm × 100 μm) and, in the next step from the colocalized NeuN und *c-fos* sections, the number of *c-fos*/NeuN+ cells (Figure 8). The mean cell counts of the ipsilateral (stimulated) sides of the coronal sections were compared between groups. For statistical analysis of the NeuN+ cells, we compared their means in the region from 0 to 100 μm to numbers in the background within one group. As such, we calculated the difference between those two means and used its 95% CI as significance marker. The same analysis was performed for double-stained *c-fos*/NeuN+ cells. Insignificant differences revealed themselves by a 95% CI value overlapping 0 (*p* > 0.05). A 95% CI not including 0 was taken as a sign of significant differences between close by and background tissue (*p* < 0.05).

For a secondary comparison between groups, we used the software JMP (JMP 13.1.0, SAS Institute Inc., SAS Campus Drive, Cary, NC, United States) and applied a one-way ANOVA and the Scheffé method for *post hoc* testing. We defined a level of *p* < 0.05 as statistical significance.

### GFAP and ED1 Immunofluorescence Staining

**FIGURE 4:**
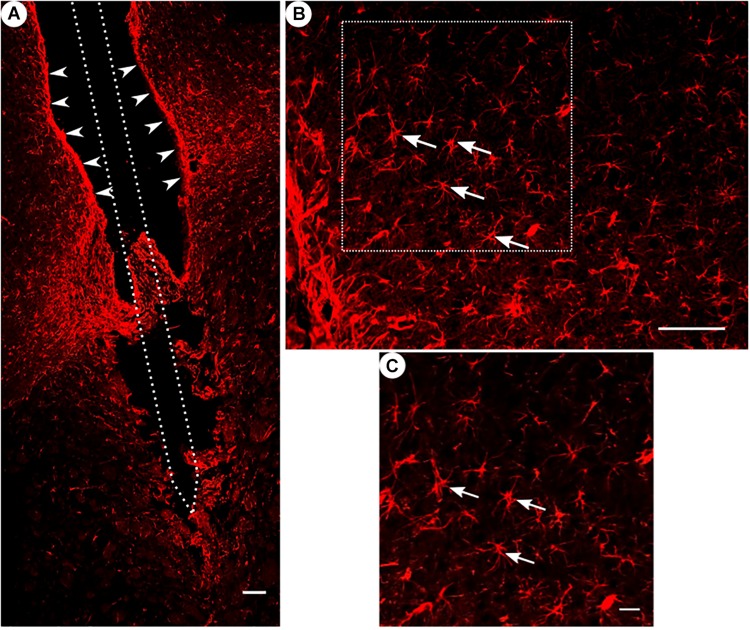
GFAP immunoreactivity. **(A)** Overview of a corticostriatal area as composite of a series of coronal sections, with arrowheads pointing at a dense glial layer at the brain tissue/microelectrode interface. Scale bar = 100 μm. **(B)** Corresponding magnified picture from panel **(A)**, white arrows pointing at GFAP+ star-shaped cells (astrocytes). Scale bar = 100 μm. **(C)** Corresponding magnified picture from panel **(B)**, with white arrows pointing at GFAP+, star-shaped cells (astrocytes). Scale bar = 25 μm.

To visualize the glial cell and microglial response, we also performed a double-label immunofluorescence staining for GFAP (Figure 4) and anti-CD68 (ED1, Figure 7). The coronal brain sections were processed and incubated for 3 h with polyclonal mouse anti-rat-CD68-antibody (AbD Serotec, United Kingdom, diluted 1:100). After rinsing in PBS, sections were incubated with a fluorescent donkey anti-mouse IgG conjugated with Alexa Fluor 488 (Abcam, Burlingame, CA, United States, diluted 1:1000). For visualizing GFAP immunoreactivity, sections were rinsed again in PBS, blocked with 10% NDS, and incubated for 3 h with a polyclonal rabbit anti-GFAP antibody (GFAP, Millipore Cooperation, Burlington, MA, United States, diluted 1:1000). After rinsing in PBS, sections were incubated with a fluorescent donkey anti-rabbit IgG conjugated with Alexa Fluor 647 (Abcam, Burlingame, CA, United States, diluted 1:1000). Finally, sections were rinsed again in PBS, mounted with DAPI-Fluoromount G (Southern Biotechnology Associates, Inc., Birmingham, AL, United States) and stored at 4°C.

### GFAP and ED1 Analysis

**FIGURE 5:**
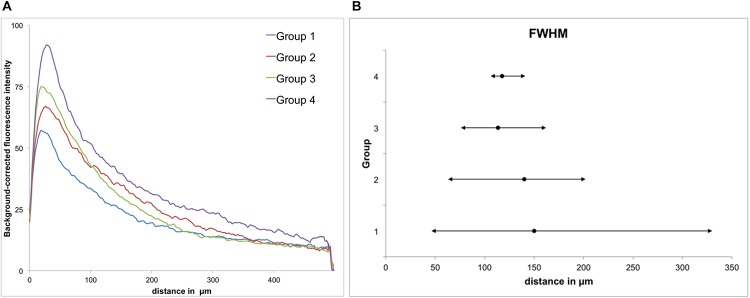
GFAP immunoreactivity. **(A)** Background-corrected mean GFAP fluorescence intensities illustrated as a function of distance from the scar’s rim for each group. **(B)** Full widths at half maximum (FWHM) of groups 1–4. Mean + maximum/minimum.

**FIGURE 6:**
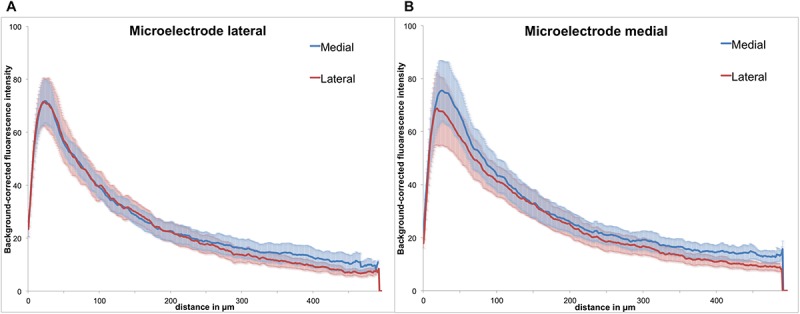
Background-corrected GFAP immunoreactivity relative to the microelectrode assembly. **(A)** Lateral from assembly. **(B)** Medial from assembly, Means ± SE.

**FIGURE 7:**
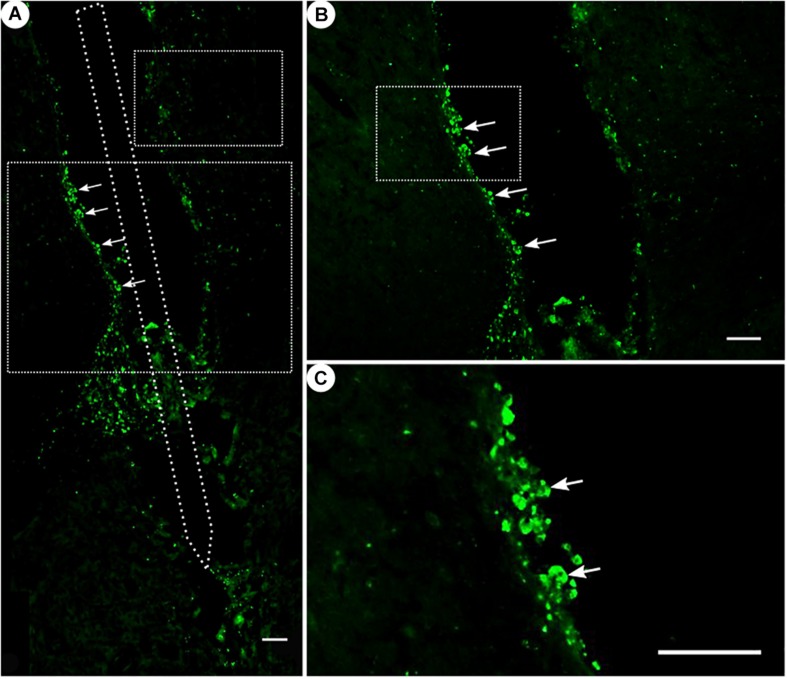
ED1 immunoreactivity. **(A)** Overview of a corticostriatal area as composite of a series of coronal sections, with arrows pointing at ED1+ cells at the tentative brain tissue/microelectrode interface. Scale bar = 100 μm. **(B)** Corresponding magnified picture from panel **(A)**, with white arrows pointing at ED1+ cells (microglia). Scale bar = 100 μm. **(C)** Corresponding magnified picture from panel **(B)**, with white arrows pointing at agglomerated ED1+ cells in the lumen. Scale bar = 25 μm.

Four coronal sections along the trajectory of each animal were used to quantify the GFAP and ED1 immunoreactivity. Images of stained sections were taken using a Zeiss microscope equipped with a ProgRes camera with ProgRes CapturePro 2.7 software (Carl Zeiss, Germany, Jenoptik, Germany). Due to the low microglial (ED1) response to the chronic implantation of the microelectrode, we could not apply a numerical analysis, and a representative picture is shown as an example (Figure 7). For quantifying the GFAP-immunoreactivity, we used ImageJ “PlotProfile” and collected several profiles for each region (cortex, corpus callosum, and striatum), separated in both medial and lateral planes of one section. We calculated the means of one region and site, and subtracted the background immunofluorescence intensities from at least 600 μm away from the scar’s rim (=background-corrected immunofluorescence intensity). The profiles of background-corrected immunofluorescence intensities of the different groups are shown in Figures 5, 6. Furthermore, we calculated the full widths at half maximum (FWHM) to quantify the expansion of the glial scar. For statistical analysis, we compared the FWHM between groups. We applied one-way ANOVA and following significant ANOVA, the Scheffé method for *post hoc* testing using the software JMP (JMP 13.1.0, SAS Institute Inc., SAS Campus Drive, Cary, NC, United States). We defined a level of *p* < 0.05 as statistical significance.

## Results

### Effects of the Chronic Implantation of a Microelectrode on NeuN Expression

Among all treatment groups, NeuN-positive cells could be found along the trajectory. They could be seen with a high density in the cortical areas as compared to the striatum (Figure 3A). Statistical analysis of NeuN+ cells in the region of 0–100 μm from the scar’s rim, the tentative former location of the implant, in comparison to a region of 400–500 μm away, showed for group 1 a 95% CI of [0.37;1.16]; group 2, [0.14;0.93]; group 3, [1.27;2.56]; and group 4, [0.37;0.95]. Thus, in all four groups, the number of NeuN+ cells in the vicinity of the tentative microelectrode was not significantly reduced as compared to background (*p* < 0.05).

### Effects of the Chronic Implantation of a Microelectrode on GFAP and ED1 Immunoreactivity

All groups had expressed GFAP alongside the former trajectory of the microelectrode, as illustrated in Figure 4A. Astrocytes agglomerated and built a dense glial layer proximal to the implant trajectory, while their typical star shape can be observed further away (Figure 4B). In Figure 5A, the background-corrected mean fluorescence intensities are illustrated as a function of distance to the implantation lesion. The highest GFAP immunoreactivity is found within a distance up to 100 μm of the scar’s rim (peak background-corrected fluorescence intensity) and decreases with increasing distance from the trajectory. The calculation of the FWHM (full width at half maximum) indicated a mean scar thickness from all groups of 129 ± 10 μm, whereas group 1 had the thickest (150 μm) and group 3 the thinnest (113 μm) FWHM (Figure 5B). Statistical analysis of the FWHM showed no significant difference between groups (*p* = 0.8826). Thus, the chronic implantation of a stiffened polyimide microelectrode leads to a reactive astrocytosis, with the formation of a glial scar with an extent of about 130 μm.

Please note in Figure 4A a fine example of a disruption of a glial sheath in the center of the picture – presumably caused by removing the implant from the wound prior to slicing.

As the polymer microelectrode was glued single sided and flat to the glass fiber support, we had essentially two different surfaces exposed back to back to the brain’s environment: polymer on the one side and silicon oxide (glass) on the other. However, when analyzing GFAP immunoreactivity with regard to the implant’s orientation, we found no discernible difference between both materials. Figure 6 illustrates the background-corrected fluorescence intensities separated in the medial and lateral directions, for both electrode materials [lateral = polymer (A), medial = glass (B)]. The results demonstrate no difference between the lateral and medial GFAP expression, with astrocytic reaction seemingly independent from the utilized material.

While ED1 expression was generally present (Figure 7), ED1-positive cells were found to form agglomerates on the scar’s edges, and could not be readily distinguished from one another.

### Effects of the Chronic Implantation of a Microelectrode on *c-fos* Expression

**FIGURE 8:**
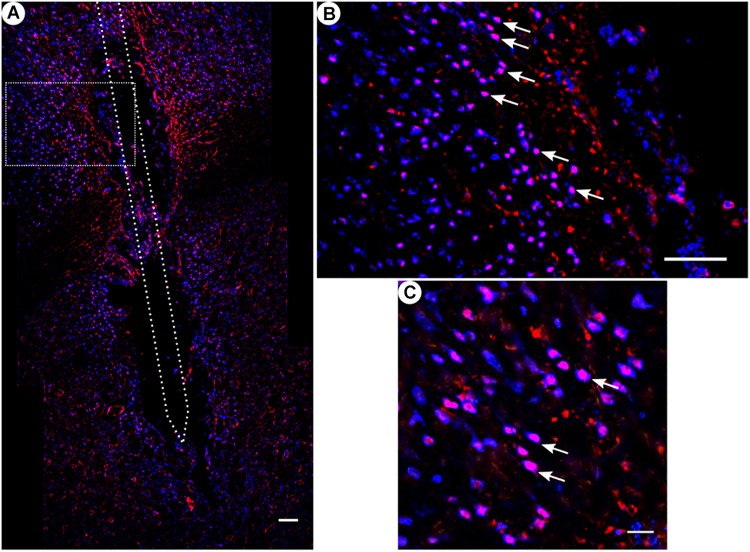
*c-fos* and NeuN immunoreactivity: **(A)** Overview of corticostriatal area as composite of a series of coronal sections, The merged image shows the colocalization of *c-fos*-labeled nuclei (red) with respect to NeuN (blue). This indicates that the induced *c-fos* activity is situated in neurons [white arrows in panels **(B,C)**]. **(B)** Corresponding magnified picture from panel **(A)**. Scale bar = 100 μm. **(C)** Further magnification from panel **(A)**. Scale bar = 25 μm.

**FIGURE 9:**
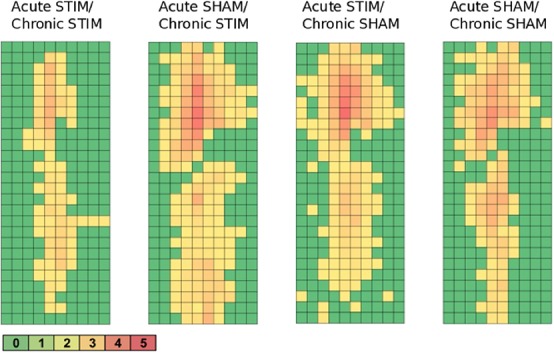
Heatmaps of *c-fos*/NeuN+ cells along the implant trajectory relative to the scar’s rim. Group 1 (Acute STIM/Chronic STIM) *n* = 4, Group 2 (Acute SHAM/Chronic STIM). *n* = 3, Group 3 (Acute STIM/Chronic SHAM) *n* = 5, Group 4 (Acute SHAM/Chronic SHAM) *n* = 3. Lookup table showing number of *c-fos*/NeuN+ cells per box (100 μm × 100 μm). See Supplementary Figure S1 for illustration.

All animal groups, independent of their stimulation paradigm, had displayed colocalized *c-fos*/NeuN+ cells along the microelectrode trajectory. Figure 8 displays the colocalization of the *c-fos*-labeled cells to NeuN-labeled neurons. Statistical analysis of *c-fos*/NeuN+ cells in the region of 0–100 μm from the scar’s rim, in comparison to the region of 400–500 μm, showed for group 1 a 95% CI of [12.43;31.46]; group 2, [38.24;66.21]; group 3, [45.27;77.61]; and group 4, [27.63;66.93]. Thus, in all four groups, the number of *c-fos*/NeuN+ cells in the vicinity of the implant’s scar was significantly higher than that of the background (*p* < 0.05). This result corroborates that a chronic implantation of a stiff microelectrode for 10 weeks can cause *c-fos* expression in neurons along the implant trajectory and thus presumably indicates neuronal activation. No *c-fos*/NeuN+ cells were found contralaterally, as there was no implant (histology not shown). The relative frequency distribution of the *c-fos*/NeuN+ cells along a full trajectory is displayed by heatmaps in Figure 9.

### Effects of Chronic High-Frequency Stimulation of the Dorsolateral Striatum on *c-fos* Expression

**FIGURE 10:**
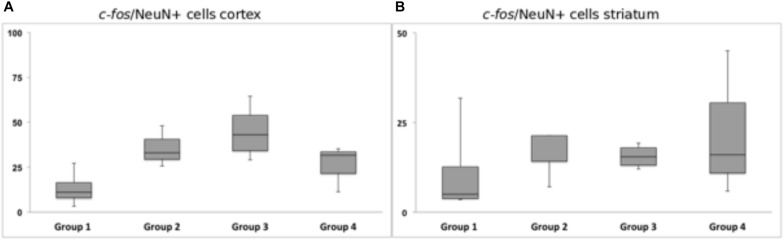
Response of *c-fos* immunoreactivity to chronic high-frequency stimulation of the dorsolateral striatum. **(A)** *c-fos*/NeuN+ cells in cortical area in a distance of 100 μm from the scar’s rim. **(B)** *c-fos*/NeuN+ cells in the striatal area in a distance of 100 μm from the scar’s rim. Whiskers showing maximum and minimum.

Chronic HFS of the dorsolateral striatum did not change the neuronal *c-fos* expression in close vicinity to the tentative microelectrode trajectory. Statistical analysis of neurons expressing *c-fos* showed no significantly reduced or higher number of *c-fos*/NeuN+ cells in the ipsilateral striatal (*p* = 0.99) and cortical (*p* = 0.12) areas. Group 1 (Acute STIM/Chronic STIM), however, did exhibit the lowest number of *c-fos*/NeuN+ cells in comparison to the other three groups (Figure 10).

## Discussion

The chronic implantation of a stiffened polyimide microelectrode assembly leads to the formation of a glial scar and an accumulation of microglia cells at the trajectory–brain interface.

In our study, we used polyimide microelectrodes glued to a 125-μm glass rod in order to achieve a precise targeting of electrical microstimulation in the dorsolateral striatum (Figure 2). After a mean implantation time of 70 days, we detected an accumulation of astrocytes (high GFAP expression) alongside the implant trajectory. The highest GFAP intensities were found within 100 μm from the scar’s rim, with a mean glial width of 130 μm, whereas the density of GFAP+ cells decreased as the distance to the implant lesion increased (Figure 5). The GFAP expression had provided no indication for a material-dependent astrocytic reaction, as the fluorescence distribution was similar in both medial and lateral directions, respectively, to PI or glass (Figure 6). Astrocytes represent 30–65% of the glial cell population in the CNS and are essential for maintaining a proper neuronal environment (Nathaniel and Nathaniel, 1981; Kimelberg et al., 1993). Following chronic implantation of an electrode, astrocytes are thought to build an encapsulation (glial scar) (Turner et al., 1999). Our findings are in accordance with reports suggesting a formation of an astrocytic boundary around the lesion, building up a tightly connected network of hyperfilamentous astrocytes, surrounded by an extracellular matrix (Bush et al., 1999; Fawcett and Asher, 1999; Faulkner et al., 2004; Seifert et al., 2006).

A significant neuronal cell loss in the vicinity of the scar’s rim was not observed. However, as can be seen in Figure 4A, the removal of the implant prior to histological preparation may have a strong detrimental impact on the integrity of the true implant/brain boundary. In fact, we recognize a strong and almost pristine appearing glial sheath in the upper, cortical part of the slice. Whereas the subthalamic region doesn’t show a closed glial sheath and instead appears ruptured. Particularly revealing seems the transition region between both, as here the structure resembles the cross-section of an inside-out turned glove’s finger, presumed to be a consequence from probe extraction.

The ED1 immunoreactivity as a result of activated monocytes and macrophages had shown a hard-to-quantify signal. Monocytes and macrophages are usually one of the first responders after an injury or lesion of the CNS and react to it by their activation (Kreutzberg, 1996). Activated monocytes begin to proliferate and change their morphology to a more “amoeboid” shape (Polikov et al., 2005). Proliferation was not observed with the residuals of the ED1+ cells (Figure 7), which were located at the tentative electrode–brain interface, the scar’s rim. Over time, the microglia’s activity and thus the acute foreign body reaction may have faded as is expected by a mean implantation time of 10 weeks (Potter et al., 2012).

The vitality of the surrounding neurons is essential for a stable signal transmission between electrode and CNS (Biran et al., 2005). The foreign body reaction, following an electrode implantation, might very well lead to neuronal cell loss and the resulting formation of a “kill zone” (Edell et al., 1992; McConnell et al., 2009a). The extent of this “kill zone” can reach up to 100 μm (Polikov et al., 2005). In our study, we could not detect a significant kill zone or neuronal cell loss in the vicinity of the trajectory’s rim. This could be either due to a weak foreign body reaction with a low ED1 expression (McConnell et al., 2009b) or simply due to a negative artifact by the destruction of the pristine probe/brain interface when removing the probe.

While *c-fos* immunochemistry is widely used as a marker for neuronal activation (Dragunow and Faull, 1989; Bullitt, 1990; Herrera and Robertson, 1996; Wilson et al., 2002), *c-fos* expression has to be considered quite unspecific regarding its mechanism of activation. *c-fos* is reported to be induced by chemical, physical, or electrical stress, and has even been found expressed in various brain regions (Greenberg and Ziff, 1984; Morgan and Curran, 1989; Hughes et al., 1992; Herrera and Robertson, 1996; Arcot-Desai et al., 2014). However, in our study, the highest density of activated neurons was located alongside the trajectory and declined with increasing distance from the implantation track (Figures 8, 9). This observation indicates that the *c-fos* expression is most likely caused by the implanted foreign body and is further supported by the low basal *c-fos* expression in the contralateral brain region. Furthermore, statistical analysis had verified the elevated number of *c-fos*/NeuN-positive cells in the vicinity of the microelectrode track, demonstrating that the implanted microelectrode resulted in an activation of the surrounding neurons. One possible explanation of this effect could be that the CNS injury caused by the implantation leads to a release of cytokines and growth factors, resulting in a modified cellular state of action (Polikov et al., 2005; He et al., 2007; Pineau and Lacroix, 2007). The cells involved in the foreign body reaction, particularly microglia and astrocytes, change their state of action in response to the trauma, and subsequently release cytokines, growth factors, enzymes, and other neuroactive substances (Eddleston and Mucke, 1993; Fawcett and Asher, 1999; Loane and Byrnes, 2010; Benarroch, 2013). Furthermore, previous evidence has demonstrated that a cortical brain injury can cause an elevated *c-fos* expression in the surrounding neurons, as a response to release of excitatory amino acids (Faden et al., 1989; Herrera and Robertson, 1990; Sharp et al., 1990). Recent studies utilizing Fast Cyclic Voltammetry (FCV) during human DBS implantations have demonstrated this “microthalamotomy” dubbed release of adenosine (Bennet et al., 2016).

Long-term HFS of the dorsolateral striatum seemed unable to significantly change the number of *c-fos*/NeuN-positive cells in the stimulated area along the lower end of the trajectory. This is surprising, as several studies report on positive effects of neuronal activation upon electrical stimulation (Krukoff et al., 1992; McKitrick et al., 1992; Arcot-Desai et al., 2014; Neyazi et al., 2016). However, differing to our HFS stimulation paradigm, the mentioned studies stimulated with low frequencies (20–40 Hz, LFS) in other regions of the brain and may thus have used a different mode of operation. Given that our chosen stimulation parameters (60 μs pulse width) coincide with known chronaxy values for neuronal fibers, but not somata, we would rather expect an axonal stimulation than a somal one (Holsheimer et al., 2000a, b; McIntyre et al., 2004; Löffler and Lujàn, 2016). As such, one would not expect a local *c-fos* expression due to electrical HFS, but rather a change in activation in fiber-connected cells. Considering the broad somatotopically organized cortico-striatal connections (Voorn et al., 2004), we looked at the *c-fos* expression of cortical neurons as well. Group 1 (Acute STIM/Chronic STIM) had shown the lowest number of *c-fos*/NeuN-positive cells, when compared to the other groups. HFS of the dorsolateral striatum might result in a suppression of the activity of cortical neurons, by means of an antidromic axonal stimulation that would affect the facilitatory GABAergic autoreceptors (Li et al., 2007; Feuerstein et al., 2011). This might help to explain an apparent reduction in *c-fos*, given that GABA acts as an inhibitory transmitter and is insensitive to *c-fos* expression (Faden et al., 1989; Hughes and Dragunow, 1995).

To conclude, our study has shown evidence that sole chronic implantation of a stiffened polyimide microelectrode (groups 1–4), and even an absence of electrical stimulation (group 4), leads to a *c-fos* expression along its trajectory.

## Data Availability Statement

The datasets generated for this study are available on request to the corresponding author.

## Ethics Statement

The animal study was reviewed and approved by the Animal Care Committee of the University of Freiburg under supervision of the Regierungspräsidium Freiburg (approval G13/97).

## Author Contributions

PP: experiments, formal analysis, visualization, methodology, and writing. RP: conceptualization, experiments, supervision, and writing. NM: methodology. UH: conceptualization, resources, supervision, funding acquisition, validation, and writing – review and editing.

## Conflict of Interest

The authors declare that the research was conducted in the absence of any commercial or financial relationships that could be construed as a potential conflict of interest.

## Supplementary Material

FIGURE S1 Artistic sketch demonstrating one exemplary composite *c-fos*/NeuN+ IHC with an approximated 100µm counting grid on top. Count results from these grids are shown in Figure 9 heatmaps. The sketch includes the targeted coordinate in the striatum (red cross) and the corresponding excerpt from the rat atlas (Paxinos and Watson, 2007).
