# Supplementary figures and images for: Chronically Implanted Microelectrodes Cause c-fos Expression Along Their Trajectory
Source: Front Neurosci. 2020 Jan 10;13:1367. doi: 10.3389/fnins.2019.01367 (PMC6965008; doi:10.3389/fnins.2019.01367)

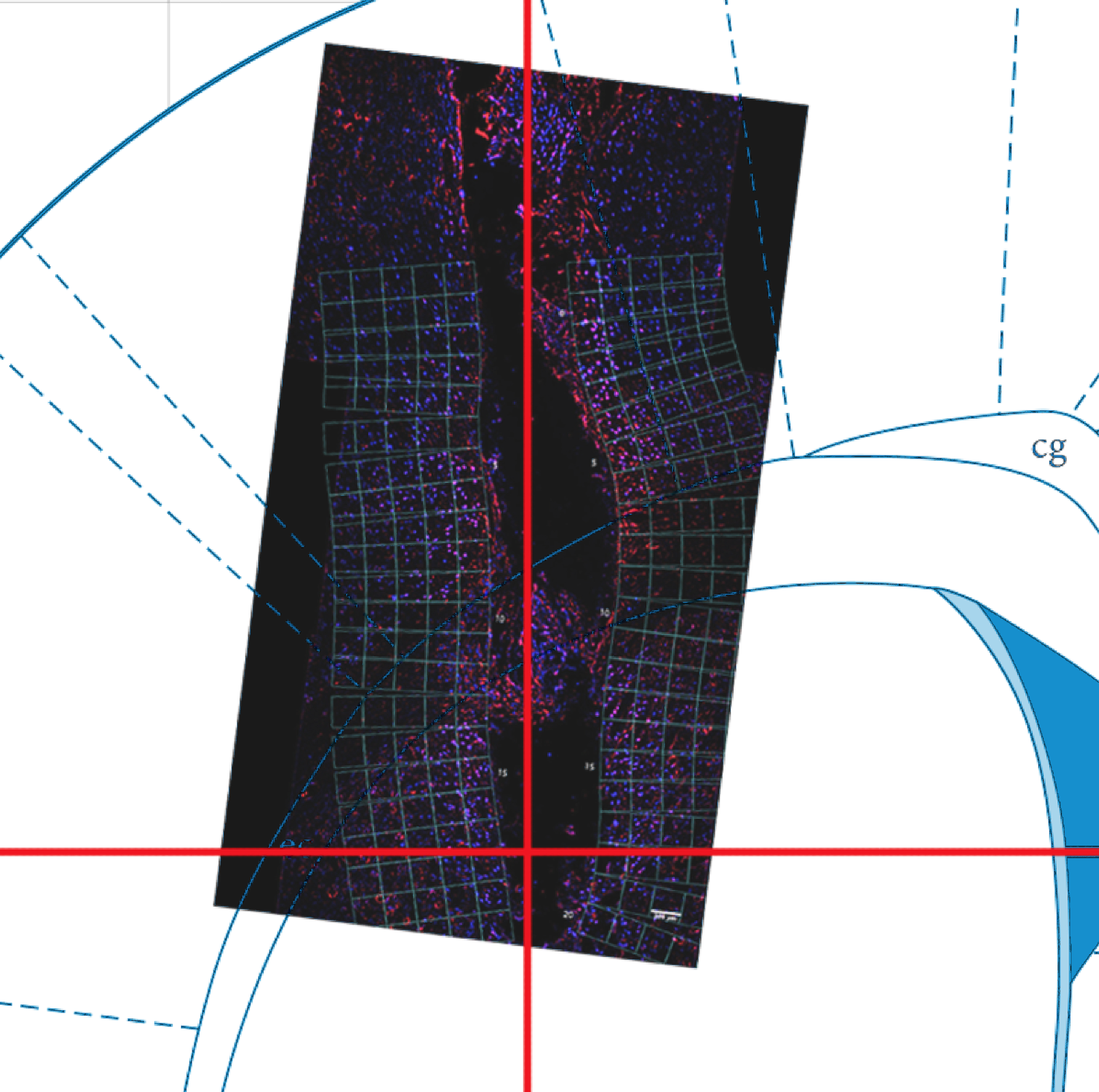

Supplement: FIGURE S1 — Artistic sketch demonstrating one exemplary composite c-fos/NeuN+ IHC with an approximated 100µm counting grid on top. Count results from these grids are shown in Figure 9 heatmaps. The sketch includes the targeted coordinate in the striatum (red cross) and the corresponding excerpt from the rat atlas (Paxinos and Watson, 2007). [file Image_1.png]
